# PI(3,4)P2 Signaling in Cancer and Metabolism

**DOI:** 10.3389/fonc.2020.00360

**Published:** 2020-03-31

**Authors:** Luca Gozzelino, Maria Chiara De Santis, Federico Gulluni, Emilio Hirsch, Miriam Martini

**Affiliations:** Department of Molecular Biotechnology and Health Sciences, University of Torino, Turin, Italy

**Keywords:** AKT, INPP4, PTEN, cancer biology, phosphatases, phosphoinositide, PI3K, cancer metabolism

## Abstract

The phosphatidylinositide 3 kinases (PI3Ks) and their downstream mediators AKT and mammalian target of rapamycin (mTOR) are central regulators of glycolysis, cancer metabolism, and cancer cell proliferation. At the molecular level, PI3K signaling involves the generation of the second messenger lipids phosphatidylinositol 3,4,5-trisphosphate [PI(3,4,5)P3] and phosphatidylinositol 3,4-bisphosphate [PI(3,4)P2]. There is increasing evidence that PI(3,4)P2 is not only the waste product for the removal of PI(3,4,5)P3 but can also act as a signaling molecule. The selective cellular functions for PI(3,4)P2 independent of PI(3,4,5)P3 have been recently described, including clathrin-mediated endocytosis and mTOR regulation. However, the specific spatiotemporal dynamics and signaling role of PI3K minor lipid messenger PI(3,4)P2 are not well-understood. This review aims at highlighting the biological functions of this lipid downstream of phosphoinositide kinases and phosphatases and its implication in cancer metabolism.

## Introduction

**Figure 1:**
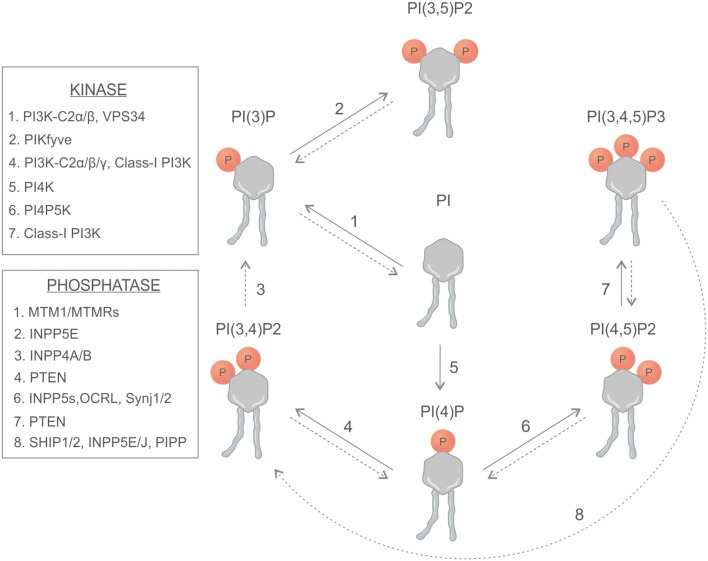
Schematic representation of phosphoinositides, kinases, and phosphatases involved in the generation of phosphoinositides.

**Figure 2:**
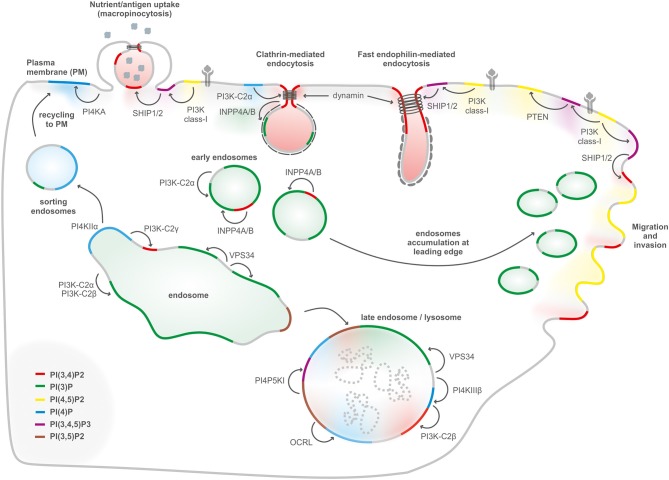
PIs conversion in internalization, migration, and endolysosomal system. Internalization of receptors and molecules through clathrin-mediated endocytosis, micropinocytosis, and fast endophilin-mediated endocytosis depends on plasma membrane PI(3,4)P2, generated either by dephosphorylation of PI(3,4,5)P3, or by synthesis from PI(4)P. PI(3)P, a key determinant of early endosomes, is generated primarily by the class III PI3K Vps34 complex II with a possible contribution of class II PI3Ks. Endosomal recycling to the cell surface generation of PI(4)P by PI4KIIα to enable exocytosis. During endosomal maturation into late endosomes, the PI(3)P 5-kinase PIKFYVE converts PI(3)P into PI(3,5)P2. The lysosomal membranes contain several PIs such as PI(3)P, PI(4)P, and PI(4,5)P2. PI(3)P can be produced by class III PI3K/Vps34 at the lysosome, PI(4)P is generated by PI4KIIIβ, and PI(4,5)P2 is hydrolyzed by OCRL. PI(4)P can be converted to PI(3,4)P2 by the class II PI3KC2β. PI(3,4,5)P3-derived PI(3,4)P2 regulates podosomes, lamellipodia, and invadopodia at the leading edge in cell migration.

Phosphoinositides are synthetized in the endoplasmic reticulum by phosphatidylinositol synthase (PIS) and consist of a glycerol backbone, an inositol ring, and two fatty acid chains; in humans, these are usually enriched with stearic acid and arachidonic acid at the *sn*-*1* and *sn-2* position of their glycerol backbone, respectively (1, 2). Different phosphatidylinositol kinases can catalyze the binding of phosphate groups at the position 3, 4, or 5 of the inositol ring, while several phosphatases can specifically remove the phosphate groups (Figure 1). The different combinations of phosphorylation give rise to seven different PIs (phosphoinositides) (3). This variety of membrane signals leads to the finely tuned recruitment of different effectors at different time points, contributing to the maintenance of membrane identity and the control of signaling pathways and cytoskeletal and membrane dynamics (Figure 2). There is growing evidence that phosphatidylinositol 3,4-bisphosphate [PI(3,4)P2] is a critical second messenger in cancer, regulating vesicular trafficking, clathrin-mediated endocytosis, cytoskeletal rearrangements (lamellipodia and invadopodia), and cell metabolism [micropinocytosis and mammalian target of rapamycin (mTOR) signaling] (4).

In this review, we will discuss the specific contributions of kinases and phosphatases to PI(3,4)P2 synthesis and how they regulate PI(3,4)P2-dependent cellular functions.

## PI(3,4)P2 Generation by Kinases

The synthesis of PI(3,4)P2 can proceed *via* class I and class II phosphoinositide 3-kinases (PI3Ks) that can directly phosphorylate the 3-OH of the plasma membrane (PM) phosphoinositide PI(4)P (5).

PI3Ks are a large family of lipid enzymes that phosphorylate the 3′-OH group of PI at the plasma membrane (Figure 1). PI3K signaling encompasses the generation of phosphatidylinositol 3,4,5-trisphosphate [PI(3,4,5)P3] and PI(3,4)P2 that activate downstream effector proteins like serine/threonine kinase AKT (5, 6).

PI3Ks have been divided into three classes according to their structural characteristics and substrate specificity. Class I PI3Ks are the most commonly studied enzymes that are activated directly by cell surface receptors like receptor tyrosine kinase (RTK) and G-protein-coupled receptors (GPCRs). Besides the class I enzymes, recent studies revealed the importance of class II PI3Ks in cell proliferation, migration, and metabolism (6). Class III PI3K consists of a single catalytic vacuolar protein-sorting defective 34 (Vps34) subunit that generates only PI(3)P, an important regulator of membrane trafficking and mTOR signaling mediator (5).

There is increasing evidence that the three class II PI3K isoforms (PI3K-C2α, PI3K-C2β, and PI3K-C2γ) have distinct and non-overlapping cellular roles. Class II PI3Ks generate PI(3)P and PI(3,4)P2 from PI and PI(4)P, respectively, on spatially defined membrane sections regulating clathrin-mediated endocytosis (7), primary cilium function (8), and insulin signaling and sensitivity (9). The three isoforms of class II PI3Ks are homologous in sequence but differ in catalytic activities and biological functions. In particular, PI3K-C2α and PI3K-C2β are expressed in a wide range of tissues where they are catalytically active in several subcellular compartments, differently from PI3K-C2γ isoform that is present in a restricted number of tissues (6).

Several papers showed a dose-dependent effect of PI3K-C2α on proliferation (10, 11), emerging as the first tumor suppressor of the PI3Ks in breast cancer (11). Increased levels of PI3K-C2β expression promote tumorigenesis in breast, ovarian, prostate neuroblastoma, and esophageal cancers, possibly by an AKT-dependent mechanism (12–15). Moreover, PI3K-C2β expression is associated with proliferation and invasion in breast cancer, being highly expressed in lymph nodes metastases compared to matching primary tumors, suggesting a pivotal role in the metastatic process.

**Figure 3:**
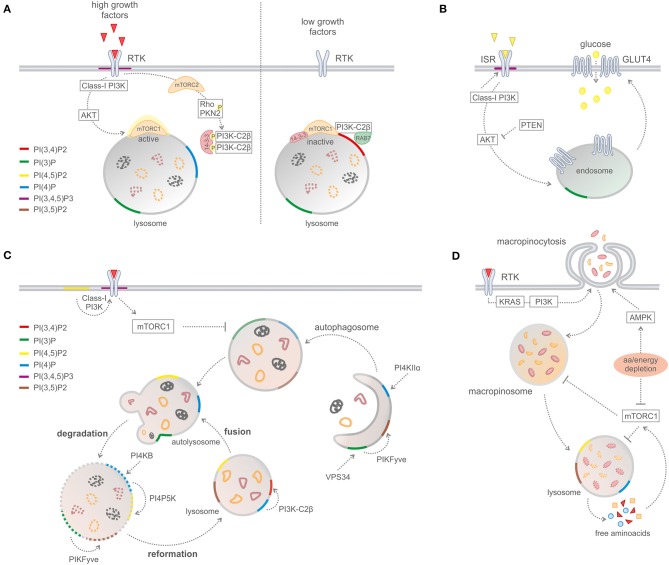
Phosphoinositides in metabolic processes. **(A)** PI3K-C2B-mediated mTORC1 regulation by PI(3,4)P2 production on lysosomes. **(B)** Glucose uptake and insulin signaling. **(C)** Macro-autophagy and recycling cytosolic components. **(D)** Macropinocytosis.

A recent study reported that PI3K-C2β represses mTORC1 activity at lysosomes by the synthesis of a specific subcellular pool of PI(3,4)P2 (Figure 3) (16). Upon depletion of growth factors, PI3K-C2β represses mTORC1 activity through the association of 14-3-3 proteins with the Raptor subunit of mTORC1 (16). In addition, the same group reported that protein kinase N (PKN) directly phosphorylates PI3K-C2β to induce its association with inhibitory 14-3-3 proteins, thus facilitating mTORC1 signaling (17).

## PI(3,4)P2 Generation by Phosphatases

### 5-Phosphatases: SHIP and INPP5

In addition to kinases, the PI(3,4)P2 levels are also controlled by specific phosphatase enzymes that hydrolyze PI(3,4,5)P3 (Figure 1). In cancer, the activity of SHIP1 and SH2-containing inositol 5′-polyphosphatases (SHIP2) and 3-phosphatase tensin homolog (PTEN) is generally considered as a negative regulator of the PI3K axis by reducing the PI(3,4,5)P3 levels at the plasma membrane. The SHIP family includes two gene products, SHIP1 (encoded by *INPP5D* gene) and SHIP2 (*INPPL1* gene), that share 43% of sequence identity at the amino acid level. They are composed of a SH2 domain at their N-terminal region, a 5-phosphatase domain and a prolin-rich C-terminal region that can be cleaved, generating different SHIP isoforms (18). SHIP acts as a specific phosphoinositide 5-phosphatases that dephosphorylates the PI(3,4,5)P3 to produce PI(3,4)P2 (19). SHIP1 expression is mainly restricted at the hematopoietic compartment, where it plays a key role in cytokine signaling, whereas SHIP2 has a wider expression pattern, including in the skeletal muscle, the heart, and the pancreas (20). SHIP1/2 are known to act as tumor suppressors, being frequently down-regulated in several types of cancer, and they counteract the PI3K pathway by producing PI(3,4)P2 (21, 22). Nevertheless, there is growing evidence indicating that PI(3,4)P2 is critical for AKT activation, thus suggesting that SHIP1/2 have a proto-oncogenic activity. Recently, pan and selective SHIP chemical inhibitors have been identified for the treatment of hematological malignancies (Table 1). In particular, SHIP1-selective chemical inhibitor 3AC (3α-aminocholestane) is able to block multiple myeloma (MM) cell lines, inducing apoptosis, and cell cycle arrest. Interestingly, SHIP inhibition can be rescued by the addition of exogenous PI(3,4)P2, suggesting that these inhibitors may have a broader application in multiple tumor types other than MM (23).

**Table 1:** Summary of main PI(3,4)P2 sources, localization, biological localization, implication in cancer, and main inhibitors of the kinase/phosphatase implicated.

**Lipid product**	**Kinase/phosphatase**	**Localization**	**Role in cancer**	**Type of cancer**	**Inhibitors**
PI(3,4)P2	PI3Kα	PM	Proliferation, metastasis	GBM, BC, CRC, gastric cancer, AML, hepatocellular carcinoma, and lung cancer	LY294002 Wortmannin, Burparlisib, Pictilisib, PQR309, Dactolisib, Apitolisib, Taselisib, Copanlisib, Alpelisib, GSK2636771
	PI3Kβ	PM	Proliferation, migration, macropinocytosis	BC	
	PI3K-C2α	clathrin coated pits, EE	Insulin signaling, clathrin mediated endocytosis, primary cilium signaling,	BC	N/A
	PI3K-C2β	LE/lysosome	Anabolic processes, lipid uptake, invasion	Ovarian, prostate and cervical cancer, neuroblstoma	PI701
	PI3K-C2γ	EE	Glycogen synthesis, insulin signaling	N/A	Compound 26
PI(3,4)P2	SHIP	PM	Macropinocytosis, fast endophilin-endocytosis, migration	BC, colorectal and NSCL cancer, hepatocellular carcinoma, hemathological malignancies	3α-aminocholestane
PI(3)P	INPP4A	EE	Proliferation, metastasis	Pancreatic cancer, lung cancer, hepatocellular, bladder and oesophageal adenocarcinoma, ovarian cancer and melanoma	N/A

*PM, plasma membrane; EE, early endosome; LE, late endosome; GBM, glioblastoma; BC, breast cancer; CRC, colorectal cancer; AML, acute myeloid leukemia; NSCL, non-small cell lung*.

INPP5E has a substrate specificity similar to that of SHIP, being a 5-phosphatase which dephosphorylates PI(3,4,5)P3 and PI(4,5)P2 (24). INPP5E has a broad pattern of expression and it was reported to negatively regulate insulin-like growth factor 1-mediated AKT phosphorylation, counteracting the PI3K axis. Similarly, INPP5K, also known as skeletal muscle and kidney-enriched inositol 5-phosphatase (SKIP), suppresses insulin signaling in the skeletal muscle in an AKT-dependent manner (25, 26).

Differently from SHIP which mainly dephosphorylates PI(3,4,5)P3 and PI(4,5)P2, inositol polyphosphate 5-phosphatase (PIPP) is mainly involved in the hydrolysis of PI(3,4,5)P3 to PI(3,4)P2 (Figure 1). PIPP, also named as INNP5J, has two prolin-rich domains at the N- and C-terminal, respectively, and a SKIP C-terminal homology domain. In cancer, loss of enzymatic activity of the PIPP induces PI(3,4,5)P3 accumulation, promoting AKT1-dependent tumor growth and progression (27). In contrast, PIPP overexpression in esophageal squamous cell carcinoma decreases PI(3,4,5)P3 levels and AKT phosphorylation, concomitantly suppressing cell proliferation and anchorage-independent growth (28).

### 3-Phosphatase

Among the phosphatases, PTEN is one of the major regulators of PIs metabolism in the cell. PTEN is responsible for the hydrolysis of PI(3,4,5)P3 to PI(4,5)P2. From a structural point of view, PTEN consists of a phosphatase domain that contains the active site and a C2 domain that binds to the phospholipid membrane (29). The tumor suppressor activity of PTEN is based on the reduction of PI(3,4,5)P3 levels, repressing AKT activation and regulating a variety of cellular processes including proliferation, survival energy metabolism, and cellular architecture (30). Inactivating mutations of the *PTEN* gene are frequently reported in human tumors, including genomic locus deletions, missense/nonsense mutations, promoter methylation, and regulation of oncogenic microRNAs (31, 32). In addition, germline mutations in PTEN gene are associated with hereditary tumor syndromes such as PTEN hamartoma tumor syndromes and Cowden and Bannayan–Riley–Ruvalcaba syndrome (33).

Although it is widely accepted that PTEN is the major negative regulator of the PI3K axis through PI(3,4,5)P3 hydrolysis, there is growing evidence showing that PTEN can also function as a PI(3,4)P2 3-phosphatase in normal and pathological conditions (34, 35). Malek et al. recently reported that PTEN loss alone has no detectable effect on the PI(3,4)P2 levels, while the concomitant loss of PTEN and INPP4B, a PI(3,4)P2 4-phosphatase, leads to PI(3,4)P2 accumulation and increased cell growth and migration within epidermal growth factor (EGF) stimulation (34). Similarly, PTEN deletion inversely correlates with PI(3,4)P2 levels in the EGF-stimulated mouse model of prostate cancer and human prostate and breast cancer cell lines. Interestingly, PTEN/INPP4B loss in non-transformed cells (Mcf10a) potentiates EGF-dependent AKT phosphorylation and invadopodia formation, although it has opposite effects in breast and prostate cancer cells, reducing AKT activation possibly by inhibiting class I PI3K signaling (35). Finally, PTEN loss leads to PI3K/AKT-mediated mTOR activation, regulating cell metabolism and promoting glycolysis and pentose phosphate pathway (PPP) (36, 37).

These results suggest a novel functional role for PTEN together with INPP4B, regulating PI(3,4)P2 levels upon EGF stimulation and compensating each other in cancer (34, 35). It is possible that technical difficulties related to PI(3,4)P2 measurement *in vitro*, using recombinant proteins, and *in vivo* (cellular extracts) masked this new PTEN activity. However, the functional role of PI(3,4)P2 in promoting and sustaining cancer growth in PTEN-dependent tumors requires further investigation.

### 4-Phosphatases: INPP4A and INPP4B

Inositol polyphosphate 4-phosphatase (INPP4A) and its isoenzyme INPP4B are magnesium-independent phosphatases that hydrolyze the 4-phosphate from PI(3,4)P2 to form phosphatidylinositol-3-phosphate [PI(3)P] (38–40). Recent findings report that INPP4 can also hydrolyze PI(4,5)P2 and PIP3 *in vitro* (41).

INPP4A and INPP4B consist of an N-terminal C2 domain, a PEST domain, and a C-terminal lipid phosphatase “CX5R” motif and they share about 40% of sequence homology (38, 39). In cancer, INPP4A and INPP4B are considered to act as tumor suppressors by inhibiting the PI3K/AKT signaling pathway in several tumor types including breast, ovary, lung, pancreatic, melanoma, and esophageal cancer (40, 42, 43). In basal-like breast cancer patients, reduced INPP4B expression is associated with poor survival rate. Similarly, in prostate cancer, loss of INPP4B correlates with poor prognosis and reduced time to biochemical recurrence by promoting AKT activation (44, 45). In follicular variant of papillary thyroid carcinoma, loss of INPP4B expression or catalytic activity in mice is not oncogenic *per se* (41, 46). However, depletion of INPP4B phosphatase domain in a *Pten*^+/−^ mice background results in malignant cancer and lung metastases by activating PI(3,4,5)P3-mediated AKT2 signaling. In normal tissue (41), INPP4B dephosphorylates both PI(3,4)P2 and PI(3,4,5)P3, but a concomitant loss of PTEN and INPP4B results in PI(3,4,5)P3 accumulation and enhanced AKT signaling, favoring tumor growth and progression (47).

Conversely, in acute myeloid leukemia, two recent reports showed that INPP4B over-expression correlates with poor prognosis (48, 49), suggesting that this effect is not due to the phosphatase activity of the enzyme (49). Therefore, in tumors with high INPP4B expression, the hydrolysis of PI(3,4)P2 can promote the activation of an alternative effector, such as serum and glucocorticoid-regulated kinase 3 (SGK3). In breast cancer, SGK3 is amplified and acts as PIK3CA oncogenic effector in a INPP4B phosphatase-dependent manner (50). In fact, PI(3)P binds to the SGK3 PX domain, leading to enhanced SGK3 activation and inhibition of AKT phosphorylation. INPP4B directly activates SGK3 through the hydrolysis of PI(3,4)P2, driving cell migration, anchorage-independent growth, and *in vivo* tumor development (50). Hence, the predominant mechanism by which INPP4B functions in cancer cells mostly relies on its ability to reduce PI(3,4)P2 levels. Further studies are required to better understand its oncogenic role in either promoting or inhibiting AKT activation in a specific genetic background or cellular context.

It is therefore possible that the level of activity of 4-posphatase differently affects oncogenic transformation on the basis of 5-phosphatase activity. As a result, one can hypothesize two scenarios:

(a) When the activity of 4-phosphatases is low, 5-phosphatase promotes PI(3,4)P2 accumulation, acting as pro-oncogenic enzymes.
(b) When the activity of 4-phosphatases is high, PI(3,4)P2 is rapidly converted into PI(3)P, and 5-phosphatases act as tumor suppressors.

Future work will lead to a further understanding of the nature of PI(3,4)P2 phosphatases in a specific cellular context and in cancer.

## PI(3,4)P2 in Cytoskeleton Rearrangements

Recent studies indicate that PI(3,4)P2 can promote cytoskeletal rearrangements at the plasma membrane by regulating lamellipodia maturation, podosomes, and invadopodia formation (4). Lamellipodia are actin-formed structures that protrude at the leading edge of the cell, which are essential for cell motility. Several works showed that the formation of these structures is highly dependent on the recruitment of lamellopodin to the plasma membrane (51–53). Lamellopodin promotes the growth of actin filaments by regulating the proteins of the enabled/vasodilator-stimulated phosphoprotein (Ena/Vasp) family, which have also been implicated in the interactions with talin and integrins (54). Of interest is the fact that lamellipodin contains a PH domain that mediates the binding to PI(3,4)P2, which possibly is produced by the 5-dephosphorylation of class I PI3K-derived PI(3,4,5)P3 at the leading edge (55).

SHIP2-derived PI(3,4)P2 also participates in the formation of podosomes and invadopodia (56). The primary function of these actin-rich structures is to degrade the extracellular matrix at the base of the cell to allow cell migration or to promote metastasis, respectively. In particular, mature invadopodia interact with the microtubule cytoskeleton, which delivers vesicular cargoes containing both membrane bound membrane type I-matrix metalloproteinase and soluble matrix metallopeptidases (57). Similar to the lamellipodia or the dorsal ruffles, the regulation of actin polymerization is strictly controlled by PIs. Indeed one of the main podosomal/invadopodial core proteins, tyrosine kinase substrate with 5 SH3 domains (Tks5), possesses a PH domain which binds PI(3)P and PI(3,4)P2 (58) and recruits N-Wiskott–Aldrich syndrome gene-like protein, actin-related proteins, dynamin-2, growth factor receptor-bound protein 2, and other proteins involved in the formation of podosomes. PI(3,4)P2 regulation in invadopodia is crucial in cancer invasion and metastasis. It has been demonstrated that, in breast cancer, the activation of PI3Kα or PI3Kβ is implicated in invadopodia formation and matrix degradation, following integrin signaling, by increasing the levels of PI(3,4,5)P3 used by SHIP2 as substrate to produce PI(3,4)P2 (59, 60). Moreover, decreased Tks5 expression correlates with reduction in tumor growth, metastasis, and angiogenesis *in vivo* (61). Reduced SHIP2 expression in PTEN-deficient glioblastoma cell lines is also linked to increased cell migration ability. In addition, SHIP2 regulates focal adhesion (FA) dynamics through the hydrolysis of PI(4,5)P2 to PI(4)P, inhibiting cell migration (62–64). In addition, SHIP2 generates PI(3,4)P2 at FA and invadopodia, inducing the development of mature FA, and lamellipodia extrusion (65). In contrast, increased SHIP2 protein levels correlate with lymph node metastasis, TNM stage, and reduced 5 year survival rate (66) in non-small-cell lung carcinoma and with increased cell migration and metastasis in breast cancer cells (67, 68). These findings indicate a novel mechanistic role of PI(3,4)P2 in the regulation of cell migration and invasion in cancer cells.

## PI(3,4)P2 in Nutrient Scavenging

Advances in the study of PIs led to a deeper understanding of the processes regulating insulin signaling, energy homeostasis, signal transduction, and intracellular trafficking.

### Glucose and Cholesterol Metabolism

PI3K-C2α is known to promote glucose uptake by regulating the insulin-dependent translocation of glucose transporter 4 (GLUT4) to the plasma membrane (69). In addition, the knockdown of PI3K-C2α levels results in the rerouting of the insulin signal, promoting a beta-cell switch from a glucose-responsive to a proliferative state (70). Recent work showed that reduced levels of liver-specific class II PI3K, PI3K-C2γ, induces the development of type II pre-diabetic condition by modulating an endosomal pool of PI(3,4)P2 required to sustain AKT2 activation (71). PI3K-C2γ null mice display reduced levels of liver glycogen and develop hyperlipidemia, adiposity as well as insulin resistance with age or after a high-fat diet. Conversely, SHIP2 null mice have normal levels of glucose and insulin but are resistant to weight gain in high-fat diet conditions (72). These results indicate that PI(3,4)P2 *in vivo* modulates glucose homeostasis in physiological conditions, regulating insulin secretion and glucose uptake.

Cholesterol metabolism involves multiple organelles, including the endoplasmic reticulum (ER), the Golgi apparatus, and the endosomal/lysosomal compartment, where exogenous cholesterol is catabolized. In particular, exogenous cholesterol is transported as cholesteryl ester by low-density lipoproteins, which can be internalized *via* receptor-mediated endocytosis. This endocytic pathway results in the fusion of endosomes with lysosomes, where cholesteryl ester is hydrolyzed and cholesterol is sorted to the ER or membranes. It is becoming more and more evident that these sorting routes are tightly controlled by PIs and, *vice versa*, the amount of cholesterol can regulate PIs turnover and lysosomal activity. Sterols and lipids can be moved between organelles and membranes by lipid transfer proteins in a non-vesicular way. Proteins of the oxysterol-binding protein 1 or oxysterol-related proteins (ORP) families can associate with phospholipids, uptake cholesterol, and mediate its transport toward acceptor membranes (73, 74). Moreover, ORP1L has also been demonstrated to be a cholesterol sensor that regulates the microtubule-dependent movement of lysosomes and late endosomes. Recent work demonstrated that ORP1L is able to strongly bind to PI(3,4)P2 on the surface of lysosomes and functions as a shuttle to transport cholesterol toward the ER (74). Through a structural analysis of the protein, the authors demonstrated that the binding of ORP1L to PI(3,4)P2 allows the cholesterol uptake from lysosomes, which in turn loosened the binding of ORP1L to the lysosomal membranes, promoting the detachment of the protein and the delivery of lipids to the ER. Interestingly, sorting of cholesterol from lysosomes dramatically decreased in ORP1L knockout cells or when PI3K-C2β was silenced, indicating that the two proteins cooperate in regulating the endosomal cholesterol efflux. Given that lysosomal cholesterol activates mTORC1 through solute carrier family 38 member 9 (75), it is evident that both the PI(3,4)P2 production by PI3K-C2β and the subsequent PI(3,4)P2-regulated cholesterol export from lysosomes contribute to the silencing of mTORC1.

### Macropinocytosis and Lysosomal Catabolism

In order to support tumor growth and fuel biosynthetic pathways, cancer cells are able to modulate vesicle generation from the plasma membrane processes (namely, endocytosis) to scavenge proteins and lipids from the extracellular space. Macropinocytosis is a regulated form of endocytosis in which cells can uptake nutrients and small particles from the extracellular environment fluid phase. Macropinosomes are formed by the folding back of dorsal membrane ruffles that fuse with the plasma membrane and create large endocytic structures (76). Oncogenic Ras can stimulate nutrient uptake by the upregulation of nutrient transporters to the plasma membrane and by inducing the membrane ruffles in a PI3K-dependent manner. Recently, it has been reported that Ras-transformed cells depend on macropinocytosis to internalize extracellular protein into the cell and sustain their metabolic needs (77). In particular, oncogenic Ras regulates the early events of macropinosome formation through the activation of class I PI3K and PI(3,4,5)P3 production at the plasma membrane (78). The actin-driven membrane ruffling is under control of class I PI3Ks. The local production of PI(3,4,5)P3 coordinates the activity of Rho and Arf GTPases, which organize the cortical cytoskeleton to form dorsal ruffles (Figure 2), and recruits PH-domain-containing effector molecules that shape the macropinosomes, such as AKT and a myosin-I motor protein (79). However, the closure of the dorsal ruffles to form the macropinocytic vesicle needs a sequential breakdown of PIs from PI(3,4,5)P3 (80). It has been observed that SHIP2 catalyzes the conversion from PI(3,4,5)P3 to PI(3,4)P2, leading to the recruitment of TAPP1 to the dorsal ruffles. TAPP1 is then able to recruit the actin-binding protein syntrophin (81), contributing to cytoskeletal rearrangement. However, it is also conceivable that PI(3,4)P2 could promote the recruitment of other effectors, like the SNX (sortin nexin) family proteins, to detach the nascent macropinosome from the plasma membrane, with a similar mechanism to CME. Next, INPP4B activity is required during this process to sequentially dephosphorylate PI(3,4)P2, indicating that this lipid is not only a degradation product of PI(3,4,5)P3 but has its own functions during micropynocitosis (80). The final hydrolysis of PI(3)P to PIs by the phosphatases MTM6 and MTM9 (myotubularin-related protein) is then necessary for the closure of the membrane ruffles and the completion of the pinocytic process (80).

The requirement of PI(3,4,5)P3 in macropinocytosis is demonstrated using PI3K inhibitors (82). Reducing PI(3,4,5)P3 levels, by the use of wortmannin or LY294002, can block the closure of macropinosomes before the circular dorsal ruffle formation (80). In addition, in tumors harboring PTEN loss, the expression of class I PI3Kβ is required to maintain elevated levels of micropinocytosis (83).

The fate of the extracellular macro-nutrients obtained by micropinocytosis is then regulated by mTORC1, which inhibits the lysosomal catabolism of proteins. Consistently, in amino-acid-starved conditions, the inhibition of mTORC1 sustains tumor growth by upregulating macropinocytosis and the catabolism of engulfed proteins (84). Aside from regulating the scavenging of extracellular nutrients, mTORC1 also negatively regulates autophagy in nutrient-rich conditions through the regulation of a protein complex composed of unc-51-like kinase 1, autophagy-related gene 13, and focal adhesion kinase family-interacting protein of 200 kDa (85). Therefore, mTORC1 suppresses the use of proteins as nutrients, limiting the cellular metabolic flexibility during times of nutrient abundance.

Recent studies revealed a link between lysosomal position and nutrient signaling *via* mTORC1 (86). Lysosome distribution is tightly controlled by a complex interplay between small GTPases, such as Rab7, and kinesins, that are in turn regulated by several factors including PI. In particular, under conditions of growth factor deprivation, PI3K-C2β is responsible for the production of a lysosomal pool of PI(3,4)P2 that inhibits mTORC1 and facilitates the perinuclear clustering of lysosomes (16). These findings suggest that the lysosomal generation of PI, including PI(3)P and PI(3,4)P2, depends on nutrient levels, directly regulating mTORC1 signaling.

Given the key role of lysosomes in nutrient sensing, future studies will need to address how PIs couple mTORC1 regulation *via* the autophagy/lysosome pathway.

### Clathrin-Mediated Endocytosis

Besides micropinocytosis, which is a clathrin- and caveolin-independent endocytotic process, CME is one of the main processes through which cells internalize surface molecules and proteins, including receptors, nutrients, and growth factors. The initialization and the timing of CME are highly regulated by a close interplay between the different PI3Ks and phosphatases, which modify the composition of the plasma membrane in the endocytic pits and recruit several effectors, such as adaptors, membrane-deforming scaffolds, and actin modulatory factors (87). For many years, PI(4,5)P2 was thought to be the main regulator of the process, given the presence of a multitude of PI(4,5)P2 binding proteins in the endocytic machinery. Early-acting clathrin adaptors AP-2 (88), membrane remodeling proteins containing BAR domains (89), and dynamin (90) are all able to bind PI(4,5)P2, giving a functional identity to the clathrin-coated pits (CCPs) and promoting the invagination and the fission of the clathrin-coated vesicles. However, it has been recently demonstrated that PI(3,4)P2 also exerts a crucial role in the maturation of the vesicles (7) (Figure 2). During the early stages of CME, different 5-phosphatases such as SHIP2 or synaptojanin are recruited to the CCP, suggesting that the levels of PI(4,5)P2 decline as the vesicle matures (91). The resulting accumulation of PI(4)P is used as a substrate for the PI3K-C2α-dependent synthesis of PI(3,4)P2. The pool of PI(3,4)P2 is able to recruit the PX-BAR domain proteins SNX9 and SNX18, which contribute to the formation of the narrow neck in the nascent vesicles that will be finally cut by dynamin, leading to the release of the vesicles (7). The production of PI(3,4)P2 also contributes to the recruitment and activation of the adaptor protein FCHSD2 (FCH and double SH3 domains protein 2) (92). This F-BAR containing protein promotes the formation of actin structures around the CCPs, leading to the efficient invagination of the plasma membrane.

Recent findings showed that PI(3,4)P2 can also be directly synthetized during the process of clathrin-mediated pinocytosis by PI3K-C2β (93). In this work, PI3K-C2α is recruited to the clathrin-coated structures through its binding to PI(4,5)P2, in a similar way to CME, while PI3K-C2β is recruited to the plasma membrane by the interaction with the scaffold protein ITSN1 (intersectin 1). The production of PI(3,4)P2 by PI3K-C2β recruits FCHSD2, which contributes to actin polymerization and drives the mechanical force needed for the abscission of the vesicle.

CME is a complex process that requires several adaptors, membrane curvature effectors, and a membrane scission machinery. Therefore, the formation of the clathrin-coated pits and the release of clathrin-coated vesicles take a relatively long time. However, in specific conditions, cells can internalize some receptors and ligand–receptor complexes in a faster way, through the so-called fast endophilin-mediated endocytosis (FEME), which does not require clathrin. This endocytic route relies on endophilin, a protein containing a SH3 domain, a BAR domain, and possesses multiple amphipathic helices (94). The coexistence of these domains allows endophilin to recognize cargo receptors by itself, to promote membrane curvature, and to support membrane scission in collaboration with dynamin (95, 96). The process of FEME starts with the activation of the plasma membrane receptors and the production of PI(3,4,5)P3. Subsequently, the production of PI(3,4)P2 by SHIP1/2 leads to the recruitment of lamellipodin and its binding partner endophilin, which induces actin rearrangement together with WASP or Ena/Vasp proteins (96) (Table 1). Thus, PI(3,4)P2 seems to act in a very similar way both in CME and in FEME.

## Role of PI(3,4)P2 in Cancer Metabolism

The formation of PI(3,4)P2 has different effects in the biology of the cell: it is an alternative pathway for the removal of PI(3,4,5)P3, a source for the production of PI(3)P, and the origin of a second messenger. The inappropriate accumulation of PI(3,4)P2 due to excess in production or defects in degradation contributes to several disorders caused by mutations in kinases and phosphatases, respectively. The main source of PI(3,4)P2 derives from the dephosphorylation of PI(3,4,5)P3 mediated by SHIP. While it is clear that the tumor suppressor function of PTEN is due to the removal of PI(3,4,5)P3 and PI(3,4)P2, the effect of SHIP on tumor transformation was debated. The tumor suppressor role of the 5-phosphatases can be associated to the removal of the PI(3,4,5)P3-dependent signaling and consequent downregulation of class I PI3K signaling or to a negative feedback loop of PI(3,4)P2 (27). On the contrary, it is now clear that, in a certain context, SHIP may facilitate tumor cell survival, contrarily to PTEN, due to a different effect on AKT. In particular, the removal of PI(3,4,5)P3 is anti-tumoral if followed by PI(4,5)P2 production mediated by PTEN, while it is pro-tumoral if it results in SHIP-mediated accumulation of PI(3,4)P2. Accordingly, the increase in PI(3,4)P2 levels, due to the loss of the tumor suppressor function of 4-phosphates, is usually considered as pro-growth (40), and mutant mice for INPP4B show mammary epithelial transformation. The SHIP-mediated increase of PI(3,4)P2 enhances the number of docking sites at the plasma membrane in order to recruit and activate PH-containing kinases such as AKT. Thus, SHIP, which is mainly expressed in blood cells, promotes MM and leukemia cell transformation. The requirement of both PI(3,4,5)P3 and PI(3,4)P2 to sustain malignant transformation is known as “the two PIP hypothesis” (18). In line with this concept, both the agonistic and the antagonistic compounds of SHIP are efficient in killing multiple myeloma cells. SHIP inhibition using 3AC abrogates MM growth (23); however, another group demonstrated that the reduction of SHIP expression levels did not affect MM cell proliferation.

While the loss of INPP4 [with PI(3,4)P2 accumulation] or SHIP [with PI(3,4,5)P3 increase] is not sufficient to promote tumor formation, PTEN deficiency alone (impacting on both lipids) is associated with a high incidence of cancer (Table 1). These data suggest that the uncontrolled production of both lipids is necessary to induce cell transformation mediated by PI3K dysregulation. Alternatively, some phosphatases may have partially redundant roles; thus, single mutants are not enough to impact on tumor onset.

Another way of PI(3,4)P2 derivation comes from class II PI3K. However, by now, there is no direct evidence about class II PI3K-derived PI(3,4)P2 in tumor. In fact, the only study based on a cancer mouse model and a patient cohort demonstrates the implication of PI3K-C2α in breast cancer progression due to its scaffold rather than to its catalytic function (11).

It is becoming clear that PI(3,4)P2 is not only a lipid component that characterizes membrane identity but that it also acts as a signaling molecule. The pathologies associated with PI(3,4)P2 accumulation are characterized not only by mutations in kinases and/or phosphatases but also by alterations in the PI(3,4)P2-dependent signaling pathway. The PI(3,4)P2 effectors contain 3-phoshoinositide binding domains, including PH, PX, FYVE, ANTH, ENTH, and FERM. Although some proteins selectively bind to PI(3,4,5)P3, most proteins, such as AKT and PDK, have dual specificities for both PI(3,4,5)P3 and PI(3,4)P2, making it difficult to discriminate between the functional contributions of the two lipids in different signaling contexts. However, it is becoming clear that PI(3,4,5)P3 mainly regulates AKT1 at the plasma membrane, while PI(3,4)P2 regulates a spatially restricted pool of AKT2 in endosomes. The AKT-mediated phosphorylation of mouse double minute 2 homolog, a negative regulator of p53, causes the activation of pyruvate kinase isozymes, which are involved in the last step of glycolysis, preventing serine synthesis and promoting glycolysis (97). By now, the only convincing specific effect mediated by PI(3,4)P2 is the Tapp1/2-induced feedback inhibition of class I PI3K. In response to insulin stimulation, the ko for Tapp1 results in increased AKT stimulation in the heart and the muscles (98). Interestingly, the same phospholipid mediates opposite effects according to its localization: at the plasma membrane, PI(3,4)P2 downstream class I PI3K activates mTOR and pro-survival signals, while on the lysosome it inhibits mTOR in serum-starved conditions. At the plasma membrane, PI3K-C2α promotes glucose uptake, impacting on GLUT4 translocation upon insulin stimulation (69).

On the contrary, another class II PI3K, PI3K-C2γ, controls the endosomal pool of PI(3,4)P2 on endosomes, where it regulates glycogen deposition in the liver. Ko mice display insulin resistance and dyslipidemia with age (71). Given its implication in metabolism and its specific pattern of expression, PI3K-C2γ could be involved in tumors highly dependent on metabolism, such as pancreatic cancer.

PI3K-C2β is also involved in the production of PI(3,4)P2 at endomembranes, in particular, on the lysosome. After nutrient deprivation, the PI3K-C2β-derived phospholipid inhibits mTOR, suggesting a metabolic impact of this kinase (16). In particular, mTORC1 activation sustains glycolysis and the PPP oxidative arm, promoting the expression of glucose-6-phosphate dehydrogenase. In addition, mTOR is responsible of the *de novo* purine synthesis for nucleic acid production and of mitochondrial biogenesis in order to boost ATP production (37, 99). It was recently demonstrated that both PI3K-C2α and PI3K-C2β regulate clathrin-mediated pinocytosis, a fluid endocytosis involved in the acquisition of nutrients from the extracellular environment (93). This study further supports the involvement of both class II PI3K in cell metabolism control.

Understanding how PI(3,4)P2 contributes to several diseases, mainly linked to cancer and metabolism, would be therapeutically beneficial. In particular, the alteration of the PI3K signaling as well as the defects in phosphoinositide-metabolizing enzymes are the main causes of PI(3,4)P2-related diseases. Given that the production of PI(3,4)P2 comes from different pathways, the manipulation of only one enzyme is not sufficient to interfere with its physiological function. By now, the main inhibitors in clinical trials target class I PI3K, while drugs against phosphatases and specific for class II PI3Ks are still at the preclinical level and need further studies.

## Author Contributions

LG, MD, FG, EH, and MM wrote the manuscript. FG did the artwork. MD made the table. All authors contributed to manuscript revision, read, and approved the submitted version.

### Conflict of Interest

EH is co-founder of Kither Biotech, a company involved in the development of PI3K inhibitors. The remaining authors declare that the research was conducted in the absence of any commercial or financial relationships that could be construed as a potential conflict of interest.
